# 

**Published:** 1870-09

**Authors:** 


VOL. XXIV. No. 9.

### THE
### Dental Register.

EDITED BY

J. TAFT & G. WATT.

SEPTEMBER, 1870.

MONTHLY:

THREE DOLLARS PER ANNUM, IN ADVANCE.

CINCINNATI:
WRIGHTSON & CO., Printers, 167 WALNUT STREET.

J. & WM. TAFT, Dentists, 117 West Fourth St.

